# Subfailure Overstretch Injury Leads to Reversible Functional Impairment and Purinergic P2X7 Receptor Activation in Intact Vascular Tissue

**DOI:** 10.3389/fbioe.2016.00075

**Published:** 2016-09-29

**Authors:** Weifeng Luo, Christy M. Guth, Olukemi Jolayemi, Craig L. Duvall, Colleen Marie Brophy, Joyce Cheung-Flynn

**Affiliations:** ^1^Department of Surgery, Vanderbilt University, Nashville, TN, USA; ^2^Department of Biomedical Engineering, Vanderbilt University, Nashville, TN, USA; ^3^VA Tennessee Valley Healthcare System, Nashville, TN, USA

**Keywords:** vascular injury, mechanical stretch, subfailure overstretch, P2X7R, vasomotor function, rat model

## Abstract

Vascular stretch injury is associated with blunt trauma, vascular surgical procedures, and harvest of human saphenous vein for use in vascular bypass grafting. A model of subfailure overstretch in rat abdominal aorta was developed to characterize surgical vascular stretch injury. Longitudinal stretch of rat aorta was characterized *ex vivo*. Stretch to the haptic endpoint, where the tissues would no longer lengthen, occurred at twice the resting length. The stress produced at this length was greater than physiologic mechanical forces but well below the level of mechanical disruption. Functional responses were determined in a muscle bath, and this subfailure overstretch injury led to impaired smooth muscle function that was partially reversed by treatment with purinergic receptor (P2X7R) antagonists. These data suggest that vasomotor dysfunction caused by subfailure overstretch injury may be due to the activation of P2X7R. These studies have implications for our understanding of mechanical stretch injury of blood vessels and offer novel therapeutic opportunities.

## Introduction

Vascular stretch injury occurs in blunt and penetrating injuries such as cervical spine hyperextension (extracranial vessel injury) and popliteal artery injury associated with knee dislocation. Surgical harvest and handling during revascularization procedures also cause overstretch vascular injury. In particular, endoscopic harvest of human saphenous vein (HSV) is associated with stretch injury and impacts the outcome of coronary artery bypass procedures (Cook et al., 2004; Rousou et al., 2009; Kiani et al., 2012; Andreasen et al., 2015). Vascular stretch injury is associated with thrombosis, spasm, intimal injury, and hyperplastic growth, and subsequently reduced blood flow to target organs (Van Belle et al., 1998; Lee et al., 2010; Wani et al., 2012).

Mechanotransduction and the signaling events associated with physiologic mechanical forces have been well characterized [reviewed in Hahn and Schwartz (2009)]. However, much less is known about the responses to surgical or traumatic mechanical stretch injury and the molecular events that transduce pathologic mechanical injury in vascular tissues. Porcine saphenous veins subjected to stretch injury during harvest have decreased vasomotor function that is ameliorated by treatment with the P2X7R antagonist brilliant blue FCF (Hocking et al., 2015). In addition, harvest and preparation of HSV leads to decreased vasomotor function that is ameliorated by brilliant blue FCF treatment (Voskresensky et al., 2014). These data suggest that injury during surgical harvest and preparation of saphenous veins is associated with vasomotor dysfunction that is restored by treatment with P2X7R antagonists (Voskresensky et al., 2014; Hocking et al., 2015). These data further suggest that pathologic surgical injury of vascular tissues may be mediated by P2X7R.

P2X7R has been shown to be activated by kidney injury (Yan et al., 2015), lung injury (Riteau et al., 2010), and neurologic injury (Yu et al., 2009). P2X7R activation leads to a variety of cellular events, including release of ATP from pores and channels (Liang and Schwiebert, 2005), influx of calcium which leads to cytolysis and release of ATP (Ballerini et al., 1996; Surprenant et al., 1996), and regulation of signaling pathways that lead to apoptosis and inflammation (Neary et al., 2003; Donnelly-Roberts et al., 2004; Lister et al., 2007). Thus, P2X7R activation is a response to injury that may potentiate that injury by further release of ATP and subsequent activation of P2X7R signaling events.

In this study, we developed and characterized the biomechanical properties and functional consequence of vascular stretch injury consistent with the injury that occurs during endoscopic harvest in a rat abdominal aorta (RA) model. The hypothesis of this investigation is that mechanical stretch injury to vascular tissue leads to the activation of P2X7R. This hypothesis poses a novel signaling mechanism, whereby mechanical injury modulates subsequent injury responses in vascular tissues. The RA model allows elucidation of molecular events associated with vascular subfailure overstretch injury. Findings from these studies may lead to the development of therapeutics to alleviate vascular overstretch injury.

## Materials and Methods

All chemicals were purchased from Sigma Chemical Co. (St. Louis, MO, USA) unless otherwise specified.

### Procurement of Human Saphenous Vein

Human saphenous vein was obtained after approval from the Institutional Review Boards of Vanderbilt University Medical Center from patients undergoing coronary artery bypass grafting procedures. Vein segments were collected immediately after initial exposure without stretch [“unprepared” (UP) vein samples] and again after harvest and graft preparation [“after preparation” (AP) vein samples]. Vein segments were transported to laboratory in heparinized PlasmaLyte at room temperature (HP; 10 U heparin/mL PlasmaLyte) for experimentation within 30 min of collection.

### Procurement of Rat Abdominal Aorta

Abdominal aorta were collected from female, 250–300 g, Sprague Dawley rats (RA). Animal procedures followed study protocols approved by the Vanderbilt Institutional Animal Care and Use Committee and adhered to the National Institute of Health guidelines for care and use of laboratory animals. Immediately after euthanasia by CO_2_ asphyxiation, the abdominal aorta was isolated *via* an incision along the mid-abdomen and *in vivo* loaded length prior to excision was measured. The RA was then excised and length recorded. The vessel was placed in HP at room temperature and transported to the laboratory for immediate testing.

### Mechanical Characterization of Longitudinal Stretch in Rat Abdominal Aorta

The abdominal aorta was chosen for this model because this segment has the highest *in vivo* axial loading along the long axis of the aorta (Kassab, 2006). RA was dissected free of fat and connective tissue. Vessels were tied at both ends using surgical sutures 10–12 mm apart and distance between the two ties was defined as the basal length (*L*_0_). The proximal end was tied to a ring support. Calibrated weights were then added progressively to the distal end, and length of the stretched tissue (*L*_1_) was measured using a hand-held digital caliber until final yielding. Yielding or failure of the vessel is defined as necking, tear, or transaction between the ties. At the end of the experiment, weight of tissue was recorded. During the test, 0.9% normal saline was sprayed in order to prevent samples dehydration. Mechanical characteristics were defined and calculated as follows:

(1) *In vivo* stretch (λ_iv_) was calculated as *in vivo* loaded length/excised length.
(2) Stretch ratio (λ_ev_) was defined as *L*_1_/*L*_0._
(3) Stress (×10^5^ N/m^2^) was defined as force (g) × 0.0987/area, where area = [wet weight (milligrams)/length (millimeters)]/1.055 (tissue density) (Khalil et al., 1998).

### Mechanical Stretch Injury and Treatment of RA

A segment of RA was reserved as non-stretched control. The remaining RA was manually stretched to 200% the excised length for 10 s and repeated twice (Hocking et al., 2011). Stretched RA was then cut into segments and incubated for 1 h at room temperature in HP with or without the P2X7R antagonists, periodate-oxidized ATP (oATP; 100 μM) or A438079 (100 μM).

Representative sampling of stress generated during stretch injury was measured by securing the proximal end to a force transducer (AD instruments model MLT1030D) interfaced with a PowerLab data acquisition system and Chart software (AD Instruments). RA was stretched to twice the length as described above.

### Measurement of Physiologic Responses

Human saphenous vein or RA (after stretch injury) was cut transversely into rings of 1–2 mm in width and suspended in a muscle bath containing a bicarbonate buffer (120 mM NaCl, 4.7 mM KCl, 1.0 mM MgSO_4_, 1.0 mM NaH_2_PO_4_, 10 mM glucose, 1.5 mM CaCl_2_, and 25 mM NaHCO_3_, pH 7.4) equilibrated with 95% O_2_/5% CO_2_ at 37°C. Force measurements were obtained using the Radnoti force transducer (model 159901A) interfaced with a PowerLab data acquisition system and Chart software. Tissues were progressively adjusted to a resting tension of 1 g for 1 h and then primed by passive stretch to 3 g and immediately returned to the resting tension. This produced the maximal force–tension relationship as previously described (Flynn et al., 2005; Voskresensky et al., 2014; Jespersen et al., 2015). After equilibration at resting tension for an additional 1 h, the rings were contract with 110 mM KCl (with equimolar replacement of NaCl in bicarbonate buffer) repeatedly until a maximum tension was obtained to determine functional viability. Contractile responses were defined by stress, calculated using force generated by tissues. Each data point was averaged from at least two rings from the same specimen.

### Statistical Analysis

Data were reported as mean responses±SEM. The statistical significance (*p* value) and achieved power of each experiment was determined using GraphPad Prism version 5.0 and G*Power version 3.1.9.2 (www.gpower.hhu.de/en.html), respectively. Paired *t*-tests were used for experiments with dependent (matched pairs) samples from the same animal. A *p* value <0.05 was considered statistically significant.

## Results

### Harvest and Preparation Impair Smooth Muscle Contractile Function of HSV

**Figure 1:**
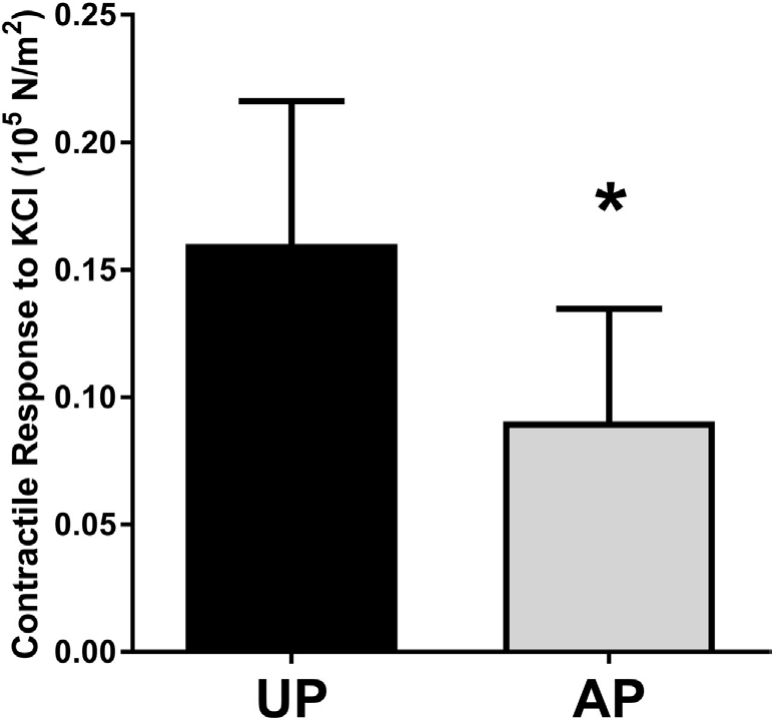
**Harvest and preparation impair smooth muscle contractile function of human saphenous veins**. Paired HSV (*n* = 6) were collected from CABG patients immediately after harvest (UP) and after graft preparation (AP) and suspended in the muscle bath. Contractile response to 110 mM KCl was measured. *p < 0.05.

To determine the impact of surgical harvest and preparation on physiologic function of HSV, segments were harvested under direct exposure with no traction (UP) or obtained after endoscopic harvest and preparation and prior to implantation (AP). Rings of 1–2 mm were suspended in a muscle bath, and smooth muscle contractile response to the depolarizing agent KCl (110 mM) was determined. Contractile responses were impaired in the AP compared to paired UP segments (Figure 1).

### Mechanical Characterization of Subfailure Overstretch Injury of RA

**Figure 2:**
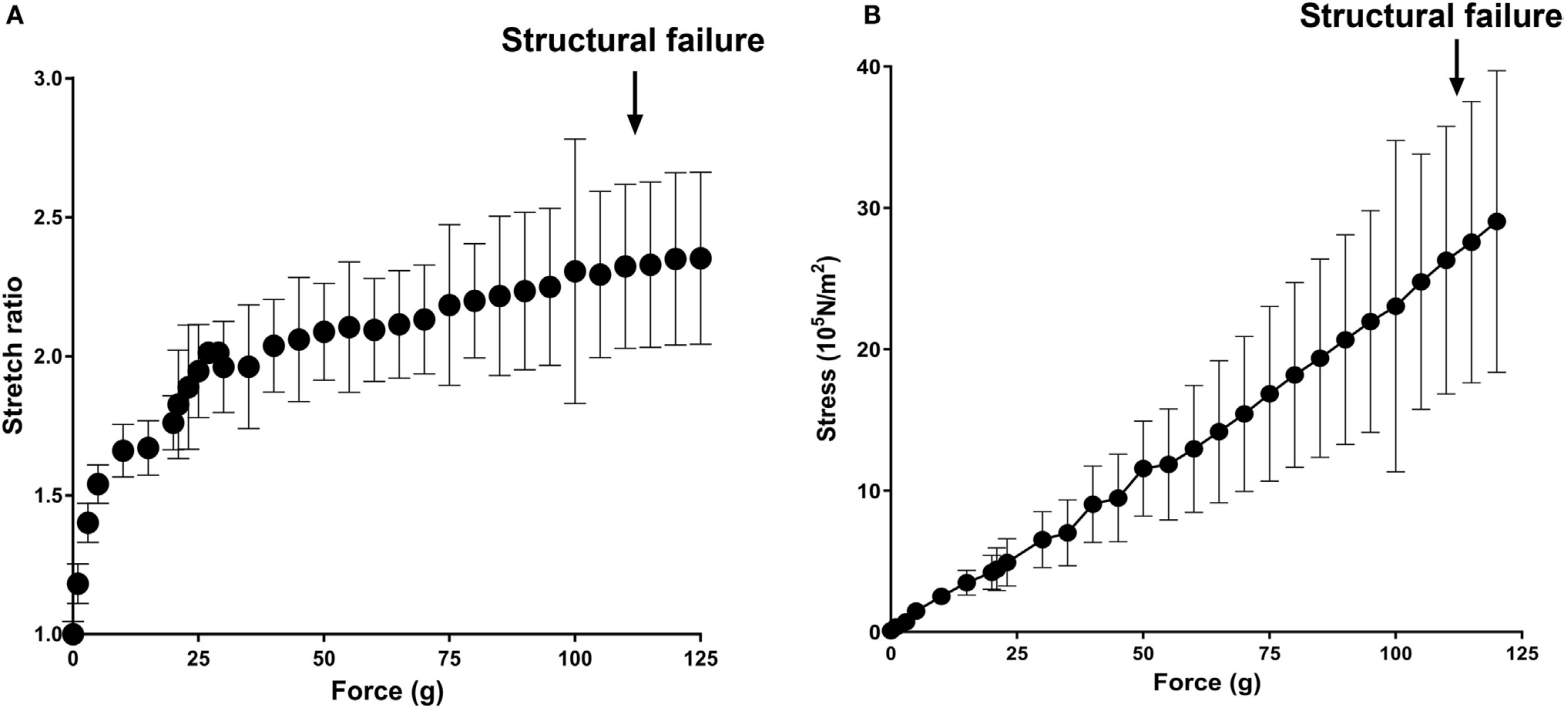
**Mechanical characterization of longitudinal stretch in the rat abdominal aorta model (RA)**. Axial load (force, g) were progressively added to stretch the RA. **(A)** Stretch ratio and **(B)** real axial stress were calculated from progressively stretched length in isolated RA (*n* = 4). Arrows indicate averaged axial load at which structural failure occurred.

**Figure 3:**
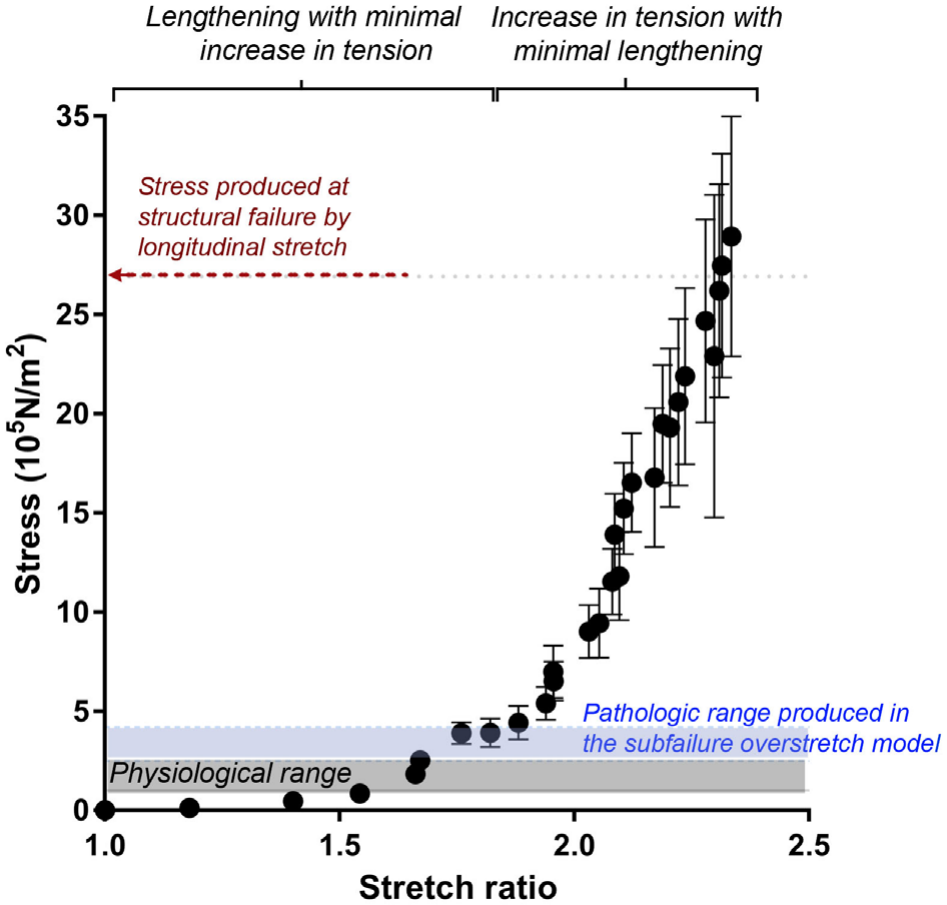
**Stretch–stress relationship of longitudinal stretch in the RA model**. Average axial stress vs. stretch ratio of progressive, longitudinally stretched RA, as shown in Figure 2, showed a non-linear mechanical behavior. Average stress produced at structural failure is indicated by the red arrow. Range of stress produced by subfailure overstretch (haptic endpoint) is indicated by blue area (*n* = 6). Gray area indicates physiological range of wall stress.

To examine vascular stretch injury under controlled conditions, we developed a traction stretch injury model using isolated RA that produced subfailure overstretch injury. First, the mechanical properties of the longitudinal stretch of isolated RA were characterized. Weights were added progressively to generate longitudinal stretch (Figure 2A), resulting in concomitant increase in stress in the tissues (Figure 2B). Stretch–stress behavior indicated that additional weight continued to increased stress with little further increase in stretch ratio until yielding of the tissue. Location of failure sites varied and structural failure occurred under 112.5 ± 33.8 g of force with a corresponding axial stress of 26.93 ± 10.92 × 10^5^ N/m^2^ (Figure 3).

**Figure 4:**
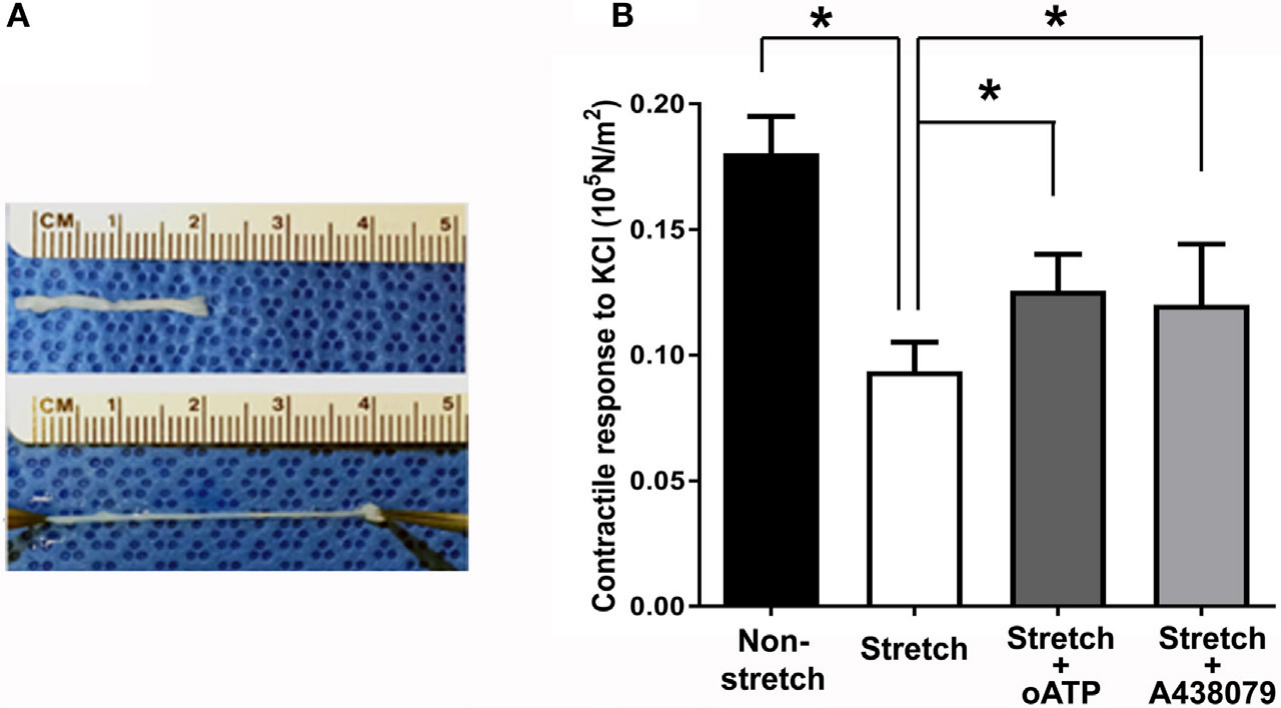
**Subfailure overstretch impaired contractility in the RA model**. **(A)** Photograph of representative overstretch injury in the RA model. **(B)** Contractile response to 110 mM KCl in RA after subfailure overstretch injury (*n* = 18). Treatment with the P2X7R antagonist oATP (*n* = 9) and A438079 (*n* = 9) restored contractile response. *n* = 9, *p < 0.05.

Next, we determined the stretch–stress behavior of RA under subfailure stretch. Isolated RA was manually stretched to the haptic endpoint, which was defined as the extent of longitudinal stretch in which the experimenters sensed force feedback of the vessels without producing structural failure (Figure 4A). λ_ev_ was produced similarly by multiple experimenters and corresponds to the maximal level of stretch that would typically occur under surgical conditions. Representative sampling (*n* = 6) of λ_ev_ during experimental traction stretch injury is 2.1 ± 0.2 (twice the excised length) with a corresponding stress of 3.26 ± 0.62 × 10^5^ N/m^2^ (Figure 3).

### Impairment of Contractile Response by Subfailure Overstretch Injury Is Reversible by P2X7R Antagonism

Subfailure overstretch impaired the contractile response of isolated RA to depolarizing KCl (Figure 4B; 0.181 ± 0.02 vs. 0.094 ± 0.05 × 10^5^ N/m^2^, *n* = 18, *p* < 0.001). We next determined the effects of P2X7R blockade on functional impairment after RA subfailure overstretch injury. Treatment of stretch injured RA with oATP, an irreversible inhibitor (Lowe and Beechey, 1982), and A438079, a highly specific tetrazole-based small molecule (Nelson et al., 2006), after stretch injury led to improvement in contractile responses (Figure 4B; 0.106 ± 0.04 vs. 0.126 ± 0.04 × 10^5^ N/m^2^ for oATP, *n* = 9, *p* = 0.012, and 0.081 ± 0.02 vs. 0.120 ± 0.02 × 10^5^ N/m^2^ for A438079, *n* = 9, *p* = 0.02).

## Discussion

Endoscopic harvest leads to traction stretch of the HSV. In addition, the HSV is prepared on the back table prior to implantation, and these preparation techniques may further injure the HSV (Eagle et al., 2011; Li et al., 2013; Wise et al., 2015). This injury is associated with functional impairment of saphenous vein (Figure 1).

To determine if P2X7R activation was associated with vascular stretch injury, a model of subfailure overstretch of rat aorta was developed. Physiological, *in vivo* axial strain on vessels is typically in the 40–80% range (Learoyd and Taylor, 1966; Guo and Kassab, 2003). Length–tension curves of isolated RA showed that the vessel lengthening with minimal increase in stress and reached a plateau at λ_ev_ = 2 at a force of 41.50 ± 21.81 g with a corresponding stress of 8.76 ± 3.76 × 10^5^ N/m^2^, far below the force that led to structural failure (Figure 2). The rapid increase in stress without noticeable lengthening of the vessel beyond λ_ev_ = 2 is due to the tensile strength of the vessels.

In the RA model, the vessel could be stretched to twice their excised length before lengthening was limited by haptic feedback. Stretch–stress curve showed that this level of stretch was just above the range of physiological axial stress but well below the range at which structural failure occurred (Figure 3) (Lawrence and Gooch, 2009; Krishnan et al., 2015). Thus, the RA model of subfailure overstretch is defined as the level of haptic feedback of increased tension at which the tissues were manually stretched to twice the *ex vivo* length. This level of stretch is consistent with that would occur during haptic feedback of routine surgical handling of vascular tissue such as endoscopic harvest of HSV.

KCl maximally depolarizes vascular smooth muscle resulting in maximal contractile forces. In the RA model, subfailure overstretch led to impaired contractile responses to KCl, suggesting that overstretch injury leads to functional impairment (Figure 4). Treatment with the P2X7R antagonists, oATP and A438079, after subfailure overstretch injury of RA partially restored contractile responses to KCl (Figure 4). Significant species differences have been reported for available P2X7R antagonists. Both antagonists used in this study, oATP and A438079, attenuated rodent and human P2X7R activation at similar sensitivity (Murgia et al., 1993; Beigi et al., 2003; Sluyter et al., 2004; Donnelly-Roberts et al., 2009). This finding suggests that impaired vasomotor responses due to subfailure overstretch injury are reversible and may be due in part to P2X7R activation.

Endoscopic harvest of HSV and overstretch injury during harvest may activate molecular events that contribute to vein graft failure. P2X7R activation potentiates the response to ATP by causing further release of ATP through P2X7R channels and pores and release of ATP from cell death due to necrosis and apoptosis (Schulze-Lohoff et al., 1998). Thus, P2X7R activation may amplify the response to release of ATP. P2X7R activation leads to multiple downstream events that may modulate the response to injury including activation of the p38MAPK pathway, thrombosis, apoptosis, and inflammation (Donnelly-Roberts et al., 2004; Burnstock, 2009; Furlan-Freguia et al., 2011; Di Virgilio, 2012). These cellular events are central to the development of intimal hyperplasia in vascular grafts and contribute to vein graft failure.

## Conclusion

A model of subfailure overstretch injury of rat aorta was developed, demonstrating that subfailure overstretch vascular injury leads to reversible functional impairment that is associated with P2X7R activation. The data in this study have implications for our understanding of pathologic stretch injury to blood vessels such as that occurs during traction injury during endoscopic harvest of HSV. The findings may be extrapolated to other clinical settings such as vascular stretch injuries associated with other vascular surgical interventions and blunt trauma to the body. This model furthers our understanding of subfailure overstretch-induced P2X7R activation and may offer novel therapeutic opportunities to ameliorate vascular overstretch injury.

### Limitations

Human saphenous veins varied due to patient demographics, but effect of graft preparation on physiologic responses was adjusted for in pair-wise comparison of segments collected from the same patients. Isolated rat arteries were used to develop the subfailure stretch injury model. There may be differences in responses of arteries from different vascular beds, differences in arteries and veins, and species differences to stretch injury; however, RA represents plentiful reproducible vascular tissue.

## Author Contributions

WL, CG, and OJ were involved in the conduct of study and collection and analysis of data; CD was involved in the interpretation of data and critical review of the manuscript; CB was involved in the design of the study, analysis and interpretation of the data, drafting and critical review of the manuscript, and obtained funding; and JC-F was involved in the design and conduct of the study; collection, management, analysis, and interpretation of the data; drafting and critical review of the manuscript; and obtained funding. All authors gave final approval of the manuscript.

## Conflict of Interest Statement

The authors declare that the research was conducted in the absence of any commercial or financial relationships that could be construed as a potential conflict of interest.
